# Analysis of Autoantibodies to 3-Hydroxy-3-methylglutaryl-coenzyme A Reductase Using Different Technologies

**DOI:** 10.1155/2014/405956

**Published:** 2014-03-05

**Authors:** Lucile Musset, Makoto Miyara, Olivier Benveniste, Jean-Luc Charuel, Alexander Shikhman, Olivier Boyer, Richard Fowler, Andrew Mammen, Joe Phillips, Michael Mahler

**Affiliations:** ^1^Department of Immunology, Université Pierre et Marie Curie (AP-HP) Pitié-Salpêtrière Hospital, Paris, France; ^2^Institute for Specialized Medicine, 4125 Sorrento Valley Blvd Suite A, Del Mar, San Diego, CA 92121, USA; ^3^Inserm, U905 & Normandie University, IRIB, 76000 Rouen, France; ^4^Department of Immunology, Rouen University Hospital, 76000 Rouen, France; ^5^Department of Research, INOVA Diagnostics, INC., 9900 Old Grove Road, San Diego, CA 92131-1638, USA; ^6^Departments of Neurology and Medicine, Johns Hopkins University School of Medicine, Baltimore, MD 21205, USA

## Abstract

Diagnostic tests are needed to aid in the diagnosis of necrotizing myopathies associated with statin use. This study aimed to compare different technologies for the detection of anti-HMGCR antibodies and analyze the clinical phenotype and autoantibody profile of the patients. Twenty samples from myositis patients positive for anti-HMGCR antibodies using a research addressable laser bead assay and 20 negative controls were tested for autoantibodies to HMGCR: QUANTA Lite HMGCR ELISA and QUANTA Flash HMGCR CIA. All patients were also tested for antibodies to extractable nuclear antigens and myositis related antibodies. To verify the specificity of the ELISA, 824 controls were tested. All three assays showed qualitative agreements of 100% and levels of anti-HMGCR antibodies showed significant correlation: Spearman's rho > 0.8. The mean age of the anti-HMGCR antibody positive patients was 54.4 years, 16/20 were females, and 18/20 had necrotizing myopathy (two patients were not diagnosed). Nine out of 20 anti-HMGCR positive patients were on statin. All patients with anti-HMGCR antibodies were negative for all other autoantibodies tested. Testing various controls showed high specificity (99.3%). Anti-HMGCR antibodies are not always associated with the use of statin and appear to be the exclusive autoantibody specificity in patients with statin associated myopathies.

## 1. Introduction

Autoantibodies are a hallmark in the diagnosis of many systemic autoimmune rheumatic diseases (SARD) including idiopathic inflammatory myopathies (IIM) (reviewed in [1, 2]). Most of those autoantibodies are directed to intracellular proteins, including nuclear and cytoplasmic antigens, and based on their specificity, autoantibodies in IIM can be grouped into myositis specific autoantibodies (MSA) and myositis associated autoantibodies (MAA) (reviewed in [1–3]). The presence of MSA and MAA has become a key feature for classification and diagnosis of IIM and they are increasingly used to define clinically distinguishable IIM subsets. Among the MSA, autoantibodies against aminoacyl-tRNA synthetases (ARS) were detected in 25–35% of IIM patients. Other autoantibodies in IIM are directed to the signal recognition particle (SRP), chromodomain helicase DNA binding protein 4 (Mi-2), SAE/small ubiquitin-related modifier (SUMO-1), MJ/nuclear matrix protein 2 (NXP2), melanoma differentiation-associated gene 5 (MDA5)/clinically amyopathic dermatomyositis p140 (CADM-140), and transcription intermediary factor (TIF1-) gamma (p155/140) [2]. Anti-Jo-1 antibodies are the most common, predominantly found in 15–30% of patients with polymyositis (PM) and in 60–70% of those with interstitial lung disease (ILD). Autoantibodies directed towards other ARS are less common, each reaching less than 5% prevalence in IIM. MSA and MAA are commonly detected using immunoprecipitation (IP) or line immunoassays (LIA) [4]. Muscle pain and weakness are common side effects of statins which are commonly used to reduce cholesterol levels. About 5% of statin users experience muscle pain and weakness during statin treatment. In 2010, antibodies to 3-hydroxy-3-methylglutaryl coenzyme A reductase (HMGCR) have been identified in patients with autoimmune necrotizing myopathies associated with statin use [5–7]. Recently, a significant difference between statin-exposed and statin-unexposed anti-HMGCR positive patients has been found [8]. Therefore, diagnostic tests are needed to aid in the diagnosis of this severe clinical condition [9, 10]. This study aimed to compare different technologies for the detection of anti-HMGCR antibodies and analyze the clinical phenotype and autoantibody profile of the patients and to investigate the epitope specificity of anti-HMGCR antibodies.

## 2. Materials and Methods

### 2.1. Sera

A total of 20 samples from myositis patients positive for anti-HMGCR antibodies (see Table 1) using a research addressable laser bead assay (ALBIA, Rouen, France) identified in a previous study [11] and 20 negative controls (age and sex matched) were collected and tested using various methods. To verify the specificity of the QUANTA Lite HMGCR ELISA a total of 824 controls were tested (for details see Section 3). Diagnoses of the patients were established based on the respective disease classification criteria and as previously described [12].

Collection of patient samples was carried out according to local ethics committee regulations. Patient data was anonymously used in keeping with the latest version of the Helsinki Declaration of human research ethics.

### 2.2. HMGCR Antigens and Western Blotting

Two different HMGCR antigens were used in the present study. The first antigen was obtained from a commercial source (Sigma) and consists of the HMG-CoA reductase catalytic domain expressed in *E. coli* and fused to GST protein with a final molecular weight of 76 kDa (including the fusion protein). The other antigen was developed at INOVA Diagnostics as follows. The HMGCR DNA was cloned into the pIEx/Bac-3 vector using Homo sapiens HMGCR and transcript variant 1 (NM_000859.2) amino acids 427–888. The clone is N-terminal 10X Histidine tagged and expressed in Sf9 cells with a molecular weight of 51 kDa. The cells were grown to 2–4 × 10^6^ cells/mL in sf900 II SFM medium and infected using 20 mL of HMGCR baculovirus per 1 L of cell culture. They were incubated at 27°C for 108 hrs while rotating at 140 rpm. The cells were harvested by centrifuging at 8000 rpm for 15 minutes using an SLC-6000 rotor. The cell pellets were washed with PBS, centrifuged at 4000 rpm and the pellets were stored at −80°C prior to extraction.

The HMGCR antigen was extracted using 1 M NaCl, 20 mM Tris, 0.25% CHAPS, and 10 mM Imidazole buffer, pH 8.0. Protease inhibitor tablets were added and the cells sonicated for 3 minutes. The sonicated mixture was centrifuged at 30,000 rpm for 30 minutes using a 50.2Ti rotor. The supernatant was collected and run over a Ni++ NTA IMAC column equilibrated on the extraction buffer. The column was washed using 1 M NaCl, 20 mM Tris, 0.25% CHAPS, and 140 mM Imidazole buffer, pH 8.0 and eluted using 1 M NaCl, 20 mM Tris, 0.25% CHAPS, and 400 mM Imidazole buffer, pH 8.0. The elution was collected and buffer exchanged using a G25 SEC equilibrated using 1 M NaCl, 10 mM Tris, 0.25% CHAPS, and 0.09% NaN3 buffer, pH 8.0. The HMGCR antigen was quantified using the calculated extinction coefficient and A280 absorbance measured using a UV/Visible spectrophotometer and stored at −80°C.

In-house antigen (lot #ALO38) was compared to Sigma antigen (HMG-CoA reductase H7039 059K4055) via SDS PAGE/western blot. Both antigens were loaded to a 15-well 4–12% Bis-Tris prepact polyacrylamide gel (Life Technologies, Carlsbad, California), at 0.5 *μ*g per well. A SeeBlue Plus2 prestained MES ladder (Life Technologies) was run in lane 1 for molecular weight determination. Electrophoresis was performed using a Mini Blot gel box and MES running buffer (Life Technologies). Proteins were run at 200 volts for 45 minutes using a BioRad Model 200/2.0 power supply.

The ladder in lane 1 was cut from the gel to be stained separately. The remaining samples were then transferred to a nitrocellulose membrane using a life technologies iBlot transfer unit. The nitrocellulose membrane was rinsed in DI water then allowed to dry. The membrane was then cut into 8 strips with each containing 1 lane of each antigen.

Strips were then incubated in HRP Sample Diluent (INOVA 508551) for 30mins followed by incubation with the appropriate patient samples at a 1 : 100 dilution for 1 hr. The strips were then washed with HRP Wash (INOVA 508552) 4 × 5 min and incubated with a goat anti-human secondary antibody diluted 1 : 3000 (Jackson Immuno Research) in HRP sample diluent for 1 hr. Strips were washed 4 × 5 min in DI water then developed with BCIP/NBT (Moss, Inc.).

### 2.3. Diagnostic Tests

#### 2.3.1. Assays for Anti-HMGCR Antibodies

The QUANTA Flash HMGCR (research use only) assay is a novel CIA that is currently used for research purposes only and utilizes the BIO-FLASH instrument (Biokit s.a., Barcelona, Spain), fitted with a luminometer, as well as all the hardware and liquid handling accessories necessary to fully automate the assay [13].

The QUANTA Flash assay for this study was developed using recombinant human HMGCR antigen coupled to paramagnetic beads. Prior to use, the reagent pack containing all the necessary assay reagents is gently inverted thirty times and the sealed reagent tubes are then pierced with the reagent pack lid. Patient serum samples are prediluted with sample buffer in small disposable plastic cuvettes. Small amounts of the diluted patient serum, the beads, and the assay buffer are all combined into a second cuvette, mixed, and then incubated for 9.5 minutes at 37°C. The magnetized beads are sedimented using a strong magnet in the washing station and washed several times followed by addition of isoluminol conjugated anti-human IgG and again incubated for 9.5 minutes at 37°C. The magnetized beads are sedimented and washed repeatedly. The isoluminol conjugate is oxidized when sodium hydroxide solution and peroxide solutions (“Triggers”) are added to the cuvette, and the flash of light produced from this reaction is measured as Relative Light Units (RLUs) by the BIO-FLASH optical system. The RLUs are proportional to the amount of isoluminol conjugate that is bound to the human IgG, which is in turn proportional to the amount of anti-HMGCR antibodies bound to the antigen on the beads.

#### 2.3.2. QUANTA Lite HMGCR

ELISA plates coated with recombinant HMGCR were incubated with diluted patient samples. Assay procedure followed standard protocol of QUANTA Lite assays (INOVA Diagnostics). A five-point calibration curve was used to convert optical density values into units. The cut-off was defined as the 99% percentile of level in a previous internal study based on disease controls.

#### 2.3.3. Quantitative Addressable Laser Bead Immunoassay (ALBIA)

The titration of anti-HMGCR antibodies was performed using a Luminex-based immunoassay, as described elsewhere [14]. Briefly, the recombinant human HMGCR catalytic domain was coupled to fluorescent BioPlex COOH-microspheres (Biorad, Hercules, CA) with the BioPlex amine coupling kit according to manufacturer's protocol. A 10 *μ*L volume containing 1,250 beads was incubated with patient's serum, in 96-well plates for 2 h. Beads were collected by filtration and washed before adding biotinylated mouse anti-human IgG Ab. After 1 h incubation and washing, anti-HMGCR autoantibodies were detected using streptavidin-R-phycoerythrin. Anti-HMGCR Ab titers were calculated from the Mean Fluorescent Intensity by comparison with a calibrator consisting in a human positive serum whose titer was arbitrarily set to 100 Arbitrary Units (A.U/mL). The threshold of positivity of this assay is 20 AU/mL.

### 2.4. Other Assays

All patients were also tested using assays for the detection of antibodies to extractable nuclear antigens (ENA, BMD, and Thermo Fisher) and myositis related antibodies (Scleroderma and myositis profile, D-Tek, Belgium).

### 2.5. Indirect Immunofluorescence on HEp-2 Slides

Autoantibodies were detected by indirect immunofluorescence on HEp-2000 cells (Reference SA2014-Ro, Immunoconcepts, Sacramento, CA, USA). Sera were tested at 1/80 screening dilution in PBS buffer, using a FITC-coupled antibody against human IgG (h + l). On these cells, the fluorescence pattern suggestive for anti-HMGCR antibodies is a finely granular cytoplasmic staining on a minority (3% or less) of cells with perinuclear reinforcement.

### 2.6. Epitope Mapping Studies

Autoantibodies to various peptides were studied using PEPperCHIP technology (PEPperPRINT GmbH, Heidelberg, Germany) [15, 16]. Peptide arrays were blocked using blocking buffer (Rockland blocking buffer MB-070 (60 min before the first assay). Sera were diluted 1 : 1000 in incubation buffer (PBS, pH 7.4 with 0.05% Tween 20 and 10% Rockland blocking buffer) and incubated for 16 h at 4°C and shaking at 500 rpm. Arrays were then washed (2 × 1 min after each assay with Washing Buffer (PBS, pH 7.4 with 0.05% Tween 20). Secondary antibody (F(ab')2 goat anti-human IgG (H + L) DyLight680) diluted 1 : 5000 was added and incubated 30 min. Identified epitopes were synthesized as soluble peptides, coated to ELISA plates and tested with anti-HMGCR positive samples. By comparing the PEPperPRINT sequence reactivity data to public domain structures of HMGCR the reactive sequences that were likely to be accessible to antibodies were determined to be ^531^GYMPIPVGVAGPL^543^, 748GYNAHAANIVTAI^760^, ^554^MATTEGCLVASTN^566^, ^689^TDKKPAAINWIEG^701^, ^561^CLVASTNRGCRAI^573^, and ^702^RGKSVVCEAVIPA^714^. Peptides were synthesized by BioSynthesis (San Diego, CA) as biotinylated constructs and tested via streptavidin ELISA assay.

### 2.7. Statistical Evaluation

The data was statistically evaluated using the Analyse-it software (Version 1.62; Analyse-it Software, Ltd., Leeds, UK). *Spearman's correlation *and *Cohen's kappa agreement test* were carried out to analyze the agreement between portions. *P* values < 0.05 were considered significant.

## 3. Results

### 3.1. Comparison of Different Antigens and Different Methods for the Detection of Anti-HMGCR Antibodies

**Figure 1:**
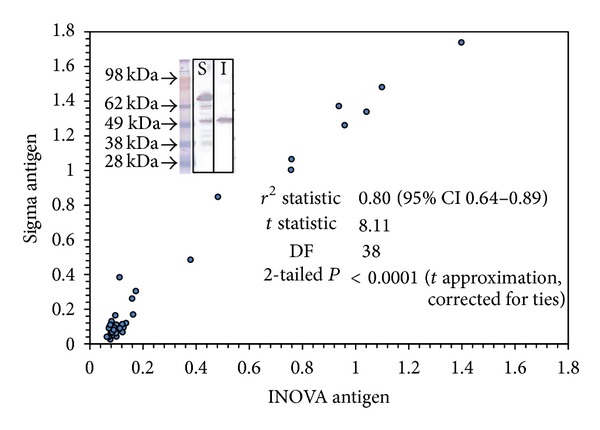
Comparison of two different antigens for the detection of anti-HMGCR antibodies using western blot and ELISA. The western blot shows the staining of a serum with anti-HMGCR antibodies of INOVA (I) and Sigma (S) antigen. A total of 40 samples with or without anti-HMGCR antibodies were compared by ELISA and the Spearman correlation shows good correlation between the two antigens.

**Figure 2:**
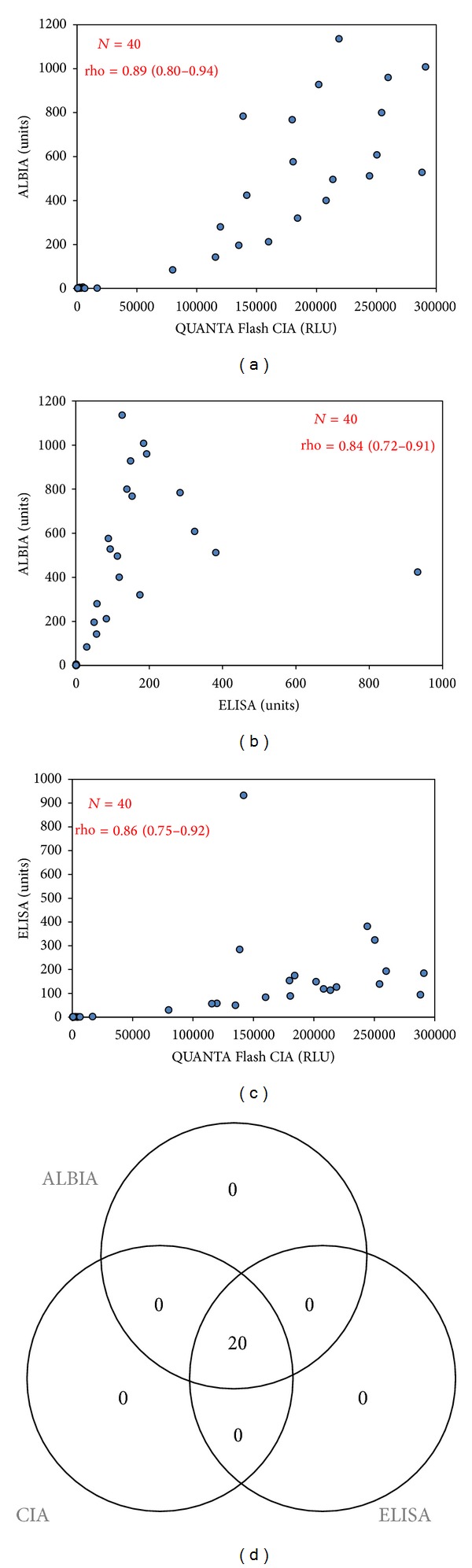
Correlation of anti-HMGCR antibodies detected using different methods. Spearman correlation diagrams are shown in (a)–(c) and a Venn Diagram in (d). All assays show good qualitative and quantitative agreements. ALBIA: addressable laser bead assay; CIA: chemiluminscent immunoassay; RLU: relative light units.

As the first step, the INOVA HMGCR antigen was compared to the Sigma antigen using western blot analysis to analyze the size, purity, and the reactivity pattern of both antigens. The western blot shows one anti-HMGCR positive sample for both INOVA and Sigma antigens. Using the Sigma antigen, several bands are stained by the serum (highest band ~75 kDa). In contrast, using the INOVA antigen, only one distinct band is recognized and stained (~55 kDa). Next, the antigens were compared by ELISA and the results obtained with the 40 samples were highly correlated (*ρ* = 0.80, see Figure 1). Subsequently, anti-HMGCR antibodies were detected using ALBIA, ELISA, and CIA and all three assays showed qualitative agreements of 100% (see Figure 2). In addition, the levels of anti-HMGCR antibodies also showed significant correlation: ELISA versus ALBIA, *ρ* = 0.84 (95% confidence interval, 0.72–0.91), ALBIA versus CIA, *ρ* = 0.89 (95% CI, 0.80–0.94), and ELISA versus CIA, *ρ* = 0.86 (95% CI, 0.75–0.92).

### 3.2. Characteristics of Patients with Anti-HMGCR Antibodies and Serological Profiling of Patients with Anti-HMGCR Antibodies

The anti-HMGCR antibody positive patients were clinically described in a previous study [11]. Briefly, their mean age was 54.4 years (range from 16 to 84; standard deviation 21.1 years) and 16/20 (80.0%) were females. In 18/20 (90.0%) of the patients, a diagnosis of necrotizing myopathy was established. The two remaining patients were not diagnosed at the last clinical follow-up but are highly suspected to suffer from myositis. The mean age at disease onset was 48.5 (SD 20.1 years, range 12–83 years). Nine out of 20 (45%) anti-HMGCR positive patients were on statin. All patients with anti-HMGCR antibodies were negative for all autoantibodies tested (SS-A, SS-B, Sm, RNP, Jo-1, Scl-70, Centromere, Mi2, PM/Scl, Ku, PL-7, PL-12, and SRP).

### 3.3. Extended Specificity Study of the ELISA and Indirect Immunofluorescence Pattern on HEp-2 Cells

**Figure 3:**
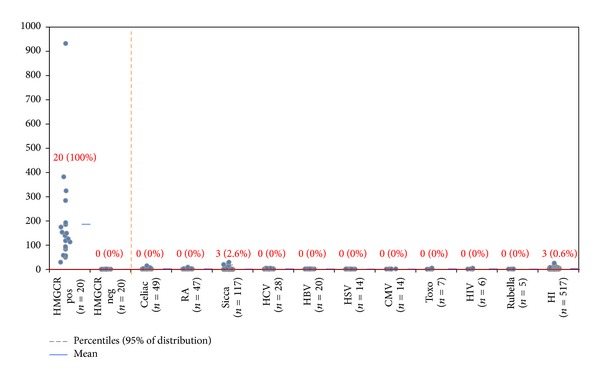
Comparative descriptive analysis of anti-HMGCR antibodies detected by ELISA. On the left side of the orange dotted line samples identified based on addressable laser bead assay are shown. On the right side of the dotted line, the results of various disease controls are displayed. Anti-HMGCR antibodies detected in various disease cohorts using ELISA show high disease specificity (99.3%).

**Figure 4:**
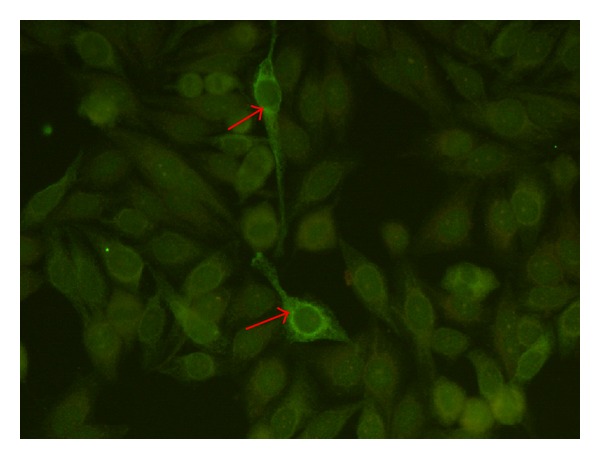
Putative indirect immunofluorescence pattern on HEp-2 cells. A serum sample from a patient with anti-HMGCR antibodies was used to stain HEp-2 cells. On these cells, the fluorescence pattern suggestive for anti-HMGCR is a finely granular cytoplasmic staining on a minority (3% or less) of HEp-2000 cells with perinuclear reinforcement.

Testing various controls showed high specificity (99.3%). 3/518 apparently healthy individuals and 3/117 patients with Sicca Syndrome were positive (see Figure 3); they all had low titers of anti-HMGCR antibodies. To investigate the staining pattern of anti-HMGCR antibodies a strongly reactive patient sample was used to stain HEp-2 cells. On these cells, the fluorescence pattern suggestive for anti-HMGCR antibodies is a finely granular cytoplasmic staining on a minority (3% or less) of HEp-2000 cells with perinuclear reinforcement (see Figure 4).

### 3.4. Epitope Mapping

**Figure 5:**
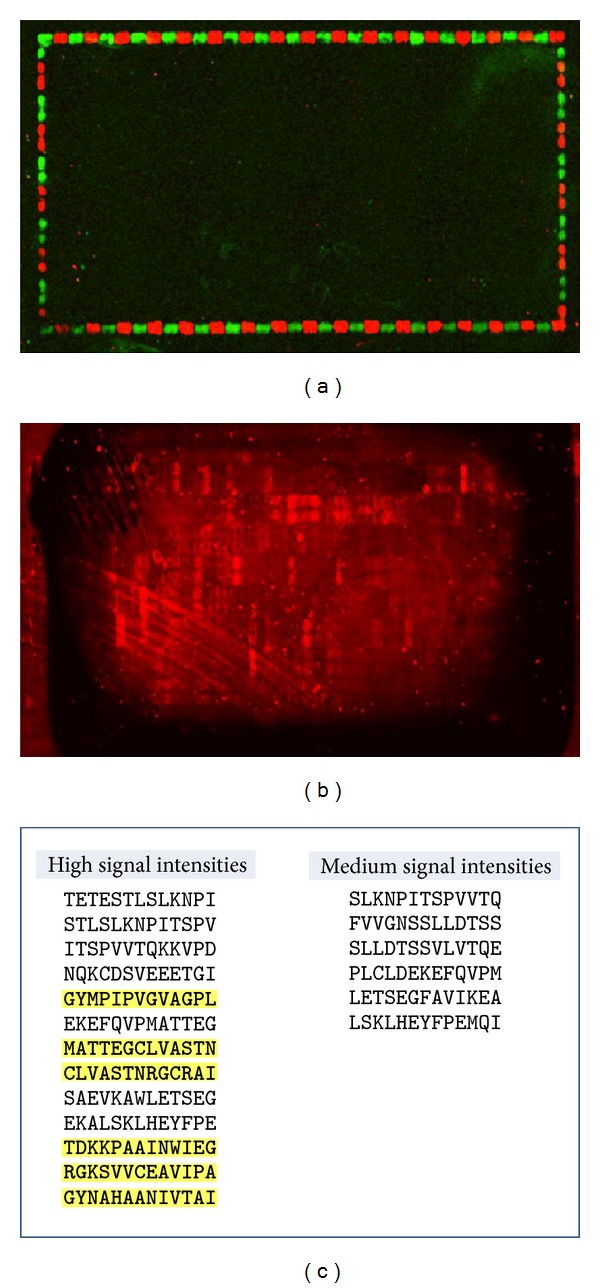
Epitope mapping of anti-HMGCR antibodies. DyLight680 showed no background interaction with the secondary antibody (a); incubation with anti-HMGCR positive serum revealed a number of reactive peptides (b). Epitopes identified using solid phase array are shown in (c). Sequences in yellow were selected for soluble peptide synthesis based on reactivity and surface exposure.

Several potential epitopes were identified using the solid phase peptide arrays (see Figure 5). A total of six sequences were selected based on surface exposure (^531^GYMPIPVGVAGPL^543^, ^748^GYNAHAANIVTAI^760^, ^554^MATTEGCLVASTN^566^, ^689^TDKKPAAINWIEG^701^, ^561^CLVASTNRGCRAI^573^, and ^702^RGKSVVCEAVIPA^714^), synthesized as soluble peptides and tested with positive sera and controls by ELISA. Reactivity found by solid phase peptide arrays could not be confirmed (data not shown).

## 4. Discussion

First described in 2010 [5], anti-HMGCR antibodies represent a promising biomarker to aid in the diagnosis and treatment decision of idiopathic inflammatory necrotizing myopathies (IINM) [1]. The present study is the first to compare different methods for the detection of anti-HMGCR antibodies (ALBIA, ELISA, and CIA), two of them manufactured in a precommercial setting and one based on ALBIA. All three assays demonstrated very good agreement. Both, the ELISA and the CIA, use a 63 kDa fragment of HMGCR which has previously been described as the epitope containing region of the antibodies [6]. In addition, we studied the coexistence of anti-HMGCR antibodies with other MSA and MAA and found that in our patients anti-HMGCR antibodies were the only detectable autoantibody. Our data on the association between statin use and anti-HMGCR antibodies confirms previous results [6]. We also confirm that the majority of patients with anti-HMGCR antibodies have IINM [5]. Anti-HMGCR antibodies might become available as a single assay on a fully automated analyzer [13], as ELISA and/or as part of multiparameter assays.

A recent study showed that the majority of patients with and without statin exposure, including those with self-limited statin intolerance, do not develop anti-HMGCR antibodies [17]. Therefore, anti-HMGCR antibodies are highly specific for those with an IIM. To further analyze the specificity especially against healthy and diseased controls, we performed a specificity study by ELISA. Using various disease controls (*n* = 824), very high specificity (99.3%) of the ELISA test was demonstrated. All six anti-HMGCR antibody positive control samples had low titers. Therefore, anti-HMGCR antibodies detected by ELISA, especially those moderate and high titers, are highly indicative of IINM. Recently, an increasing prevalence of IINM was reported. In about half of the identified patients (45%) no autoantibody could be detected. The majority of those patients were statin users (67%) compared to 18% in the group with detectable autoantibodies (i.e., Jo-1, SRP, PM/Scl, and Ro52) [18]. Future studies are mandatory to investigate the pathophysiological mechanism of anti-HMGCR antibodies and the root cause for the increasing prevalence IINM.

Although we could not find any other autoantibody in our patients with anti-HMGCR reactivity, we cannot rule out that the patients have autoantibodies that have not been tested in our study (i.e., anti-TIF1 families, anti-MDA5, anti-NXP2, and so forth).

Autoantibodies to intracellular antigens (referred to as antinuclear antibodies) are commonly tested using IIF on HEp-2 cells to aid in the diagnosis of systemic autoimmune diseases including myositis [19]. Consequently, we wanted to study if anti-HMGCR antibodies decorate certain structure on HEp-2 cells. We found that patients with anti-HMGCR antibodies frequently stain cytoplasmic structures, however, not in all cells. In this context it is important to point out that the sensitivity for antibodies to cytoplasmic antigens (i.e., Jo-1 or ribosomal P) is limited [20]. In summary, further studies are needed to (1) confirm the observed staining pattern, to (2) analyze the pattern on slides from different manufacturers and to (3) assess the reliability (sensitivity) of IIF HEp-2 for the detection of anti-HMGCR antibodies.

Using solid phase peptide synthesis, we found several peptides reacting with anti-HMGCR antibodies contained in a serum of a patient with statin-associated necrotizing myopathies. In order to confirm the reactivity using a different method, we selected surface exposed epitope sequences for synthesis of soluble peptides. Although we were unable to confirm the peptide reactivity using soluble HMGCR derived peptides, it cannot be ruled out that anti-HMGCR antibodies bind linear epitopes. However, based on our data it is likely that conformational structures play a role in the formation of the major epitope. Further studies are needed to analyze the nature of the epitope on HMGCR and to address the reason for the discrepant results between the two methods for the detection of antibodies to synthetic peptides. Different technologies including phage or bacterial display, synthetic peptides, or recombinant proteins might prove useful [19].

## 5. Conclusions

Anti-HMGCR antibodies can be detected using different methods with good intermethod agreement. Anti-HMGCR antibodies are strongly associated with IINM and to a lesser extent with statin use. Testing for anti-HMGCR antibodies might prove useful in the diagnosis of IIM and in the differentiation between self-limited and persistent statin associated myopathy which requires long-term immunosuppressive treatment.

## Figures and Tables

**Table 1:** Clinical and serological data of anti-HMGCR positive sera.

ID	ALBIA (>20)	CIA (>10,000)	ELISA (>20)	Gender	Age	Statin	ADO (ys)	CK* (25–160 U/L)	Necrosis
3	928	201891	149.2	F	62	+	59	1000	+
5	496	213681	113.5	F	53	−	52	13777	+
6	960	259814	193.1	M	59	−	47	8500	+
9	1008	291111	184.9	F	22	−	18	ND	+
10	784	138696	284.3	F	16	−	13	1500	+
11	512	244281	381.8	F	66	+	60	6500	+
13	768	179810	153.5	F	69	+	63	492	Very few fiber
15	320	184190	174.7	F	44	−	21	ND	+
17	800	254442	139.4	F	60	−	54	6000	+
18	1136	218777	126.3	F	53	+	48	4106	+
26	280	119734	57.9	M	67	+	66	5700	+
27	576	180528	88.9	F	17	−	12	17000	+
28	528	288198	93.8	M	65	−	52	500	+
29	84	79904	29.8	F	75	+	73	7429	No biopsy
30	196	135149	49.7	F	72	+	68	3700	+
31	608	250484	324.1	F	35	−	26	5700	+
32	424	141861	932.1	M	81	−	82	ND	+
34	400	208075	118.5	F	84	+	83	4119	ND
37	212	160034	83.3	F	20	−	15	612	+
39	142	115671	56.6	F	67	+	58	2000	−

CK: creatinine kinase; CIA: chemiluminescence immunoassay; ALBIA: addressable laser bead assay; ADO: age at disease onset; ND: not determined.

*Not all CK values are derived from the time point when serum was obtained for anti-HMGCR testing.

## Abbreviations

ALBIA: Addressable laser bead immunoassay
HMGCR: 3-Hydroxy-3-methylglutaryl-coenzyme A reductase
IIM: Idiopathic inflammatory myopathies
IINM: Idiopathic inflammatory necrotizing myopathies
MAA: Myositis associated antibodies
MSA: Myositis specific antibodies
SARD: Systemic autoimmune rheumatic disease
SRP: Signal recognition particle.
